# Exploring the common gene signatures and pathogeneses of obesity with Alzheimer’s disease *via* transcriptome data

**DOI:** 10.3389/fendo.2022.1072955

**Published:** 2022-12-09

**Authors:** Ting Li, Jingru Qu, Chaofei Xu, Ting Fang, Bei Sun, Liming Chen

**Affiliations:** NHC Key Laboratory of Hormones and Development, Tianjin Key Laboratory of Metabolic Diseases, Chu Hsien-I Memorial Hospital & Tianjin Institute of Endocrinology, Tianjin Medical University, Tianjin, China

**Keywords:** obesity, Alzheimer’s Disease, hub genes, weighted gene correlation network analysis (WGCNA), differential gene analysis

## Abstract

**Background:**

Obesity is a complex condition that influences several organ systems and physiologic systems. Obesity (OB) is closely linked to Alzheimer’s disease (AD). However, the interrelationship between them remains unclear. The purpose of this study is to explore the key genes and potential molecular mechanisms in obesity and AD.

**Methods:**

The microarray data for OB and AD were downloaded from the Gene Expression Omnibus (GEO) database. Weighted gene correlation network analysis (WGCNA) was used to delineate the co-expression modules related to OB and AD. The shared genes existing in obesity and AD were identified through biological process analyses using the DAVID website, which then constructed the Protein–Protein Interaction (PPI) Network and selected the hub genes by Cytoscape. The results were validated in other microarray data by differential gene analysis. Moreover, the hub gene expressions were further determined in mice by qPCR.

**Results:**

The WGCNA identifies five modules and four modules as significant modules with OB and AD, respectively. Functional analysis of shared genes emphasized that inflammation response and mitochondrial functionality were common features in the pathophysiology of OB and AD. The results of differential gene analysis in other microarray data were extremely similar to them. Then six important hub genes were selected and identified using cytoHubba, including MMP9, PECAM1, C3AR1, IL1R1, PPARGC1α, and COQ3. Finally, we validated the hub gene expressions *via* qPCR.

**Conclusions:**

Our work revealed the high inflammation/immune response and mitochondrial impairment in OB patients, which might be a crucial susceptibility factor for AD. Meanwhile, we identified novel gene candidates such as MMP9, PECAM1, C3AR1, IL1R1, PPARGC1α, and COQ3 that could be used as biomarkers or potential therapeutic targets for OB with AD.

## Introduction

Obesity is one of the most serious global public health problems. According to the World Health Organization’s (WHO) estimation, 2 billion adults worldwide are overweight or obese, of which more than 650 million are obese (1). At the current pace, an estimated 1 billion people globally, including one in five women and one in seven men, will be living with obesity by 2030 (2). Obesity is defined as an imbalance between energy intake and expenditure that leads to fat accumulation in the adipose and non-adipose tissues. Obesity is the primary risk factor for most chronic diseases, including type 2 diabetes mellitus (T2DM), dementia, liver diseases, cardiovascular diseases, numerous cancers, and so on (3–8). There is evidence that obesity could increase susceptibility to dementia (9–11). Some meta-analysis studies demonstrated that obese patients as well as those with other metabolic disorders, had a two-fold risk of developing Alzheimer’s disease (AD) (12). It is generally accepted that obesity is a state of chronic inflammation, resulting in hypertrophic adipocyte secretion of proinflammatory cytokines and adipokines that have peripheral and brain effects (13). Meanwhile, neuroinflammation is widely recognized as a hallmark of neurodegenerative diseases such as AD (14, 15). Alternatively, obesity is closely associated with the development of insulin resistance and mitochondrial dysfunction which is also considered a critical influential factor on cognitive function (16, 17). However, the molecular mechanisms linking obesity to AD remain unknown. The influence of lipid accumulation on the neurodegenerative process has not been well determined. Therefore, more studies need to be conducted.

With the rapid development of sequencing technology and bioinformatics, researchers can measure the expression of thousands of genes in various diseases. This contributes to a more thorough understanding of the pathogenesis of diseases at the genetic level. Weighted gene co-expression network analysis (WGCNA) was developed by Zhang and Horvath in 2005. Currently, WGCNA is widely used for describing the correlation patterns among genes across microarray or RNA-seq samples (18, 19). We attempted to identify gene clusters of correlating and connected shared genes in obesity and AD by WGCNA. This method has been successfully applied to identify molecular mechanisms and the risk genes associated with the phenotypes of multiple diseases.

Gene Expression Omnibus (GEO) (http://www.ncbi.nlm.nih.gov/geo/) mining identifies co-expression modules in obesity and AD. Our research demonstrated that inflammation/immune-related and mitochondrial-related genes were presented in modules highly related to obesity and AD. Inflammation pathways and mitochondrial functionality might play an extraordinary role in obesity and AD. The results were confirmed in other datasets by differential gene expression analysis. Furthermore, we verified gene expression in animals. As far as we know, this might be the first study to explore the shared gene signatures between obesity and AD using a systems biology approach.

## Materials and methods

### Data source

We used the key term “obesity” or “Alzheimer’s disease” to search OB and AD mRNA microarray datasets in the GEO database. The obtained datasets were filtered by the following criteria. First, the microarray datasets should consist of control and case groups. Second, all specimens included should be restricted to *Homo sapiens*. Third, these datasets must provide processed or raw data that could be reanalyzed. Fourth, the datasets for performing WGCNA analysis should not have fewer than 10 samples in each group. Finally, GEO datasets GSE151839, GSE118553, GSE44000, and GSE122063 were selected for further study. The data were preprocessed using the Limma package, involving background correction, normalization, and expression calculation. According to the annotation document of the corresponding platforms, the probe names were replaced with official gene symbols. The average expression level of a gene was retained if the gene corresponded to multiple probes. At last, gene expression matrix files were obtained for subsequent analyses.

### Weighted gene co-expression network analysis

Weighted gene co-expression network analysis (WGCNA) is an algorithm that can cluster genes and construct co-expressed gene modules. It is capable of exploring the relationship between gene networks and diseases. Therefore, we used the WGCNA package to build the gene co-expression networks of OB and AD. Before analysis, the Hclust function and goodSamplesGenes were performed to exclude missing and outlier samples. At first, both Pearson’s correlation matrices and the average linkage method were performed for all pair-wise genes. Then, a weighted adjacency matrix was constructed with the formula A_mn = |C_mn|^β^ (C_mn = Pearson’s correlation between Gene_m and Gene_n; A_mn= adjacency between Gene_m and Gene_n). Next, the adjacency was transformed into a topological overlap matrix (TOM) and the corresponding dissimilarity (1−TOM) which could estimate its connectivity property in the network. Average linkage hierarchical clustering was used to conduct a clustering dendrogram of the TOM matrix, and similar gene expressions were divided into different modules.

Furthermore, the correlation between the phenotype and each module was assessed. Finally, the Eigengene network was visualized.

### Identification of significant modules and functional annotation

We selected highly correlated OB and AD modules. And we uploaded the list of all genes in the OB and AD significant modules to DAVID (https://david.ncifcrf.gov/summary.jsp) for functional annotation analysis.

### Protein–protein interaction (PPI) network construction

The STRING database (https://string-db.org/) was used to construct a PPI network for analyzing protein interactions. The PPI pairs with a confidence score >0.4 were considered statistically significant, and the visualization of the PPI network of these genes was achieved by Cytoscape (version 3.9.1).

### Selection and analysis of hub genes

The hub genes were predicted using the CytoHubba. Three algorithms (MCC, MNC, and Degree) were performed to evaluate and select the hub genes. Subsequently, we constructed an interaction network of these hub genes using GeneMANIA (http://www.genemania.org/), which was reliable for discovering functionally similar genes and identifying internal relations between genes.

### DEGs and functional enrichment analysis

The R package “limma” was used to identify the DEGs between the control group and the disease group in the GSE44000 and GSE122063 datasets, respectively. The threshold of DEGs was set as *p*-value <0.05 and |logFC (fold change) | ≥0.58. Then the function and pathway of DEGs were analyzed by Gene Ontology (GO) and KEGG pathway by the R package “clusterProfiler”. The common DEGs in OB and AD were obtained using Venn.

### Animals

Eight-week-old male db/db mice, male littermate db/m mice (purchased from GemPharmatech, China), five-month-old male APP/PS1 transgenic mice, and male C57BL/6J mice (purchased from Si Pei Fu, China) were maintained at room temperature (20–24°C) and fed with a standard chow diet *ad libitum* in environmentally controlled animal facilities at the Tianjin Key Laboratory of Metabolic Diseases. Each group contained six mice. After being fed for five months, body weight was measured, and blood samples of db/db and db/m mice were collected. All mice were sacrificed by exsanguination under anesthesia with inhaled 5% isoflurane in room air. All animal procedures were approved by the Laboratory Animal Ethical Committee, Tianjin Medical University Chu Hsien-I Memorial Hospital.

### Morris water maze (MWM) test

The Morris water maze test was conducted to evaluate APP/PS1 mouse learning and memory as cognitive functions. Briefly, the MWM test was conducted in a black pool with water and a hidden platform. The pool was covered with black curtains to hide room cues and divided into the northeast, northwest, southeast, and southwest quadrants. During the orientation navigation test with four trials per day, the mice were continuously trained for 5 days. Each trial was terminated when the mice found the hidden platform, or after 60 s. The escape latency time (the time spent to find the platform) and the swim path were recorded by a camera above the task. At the end of the fifth day, the mice swam for 60 s to search for the platform after the removal of the hidden platform. The number of mice crossing the platform area and the time spent in the target quadrant were measured using the S-MART program (TECHMAN, Chengdu, China) to measure cognitive function.

### Quantitative RT‐qPCR analysis

Total RNA was extracted from subcutaneous adipose and cortex tissues separately using TRIzol reagent (Ambion). Then, complementary DNA (cDNA) was synthesized using the reverse transcription kit (TRAN, AT301-03) *via* the manufacturer’s protocol at 42°C for 15 min and 85°C for 5 s. Subsequently, messenger RNA (mRNA) levels were assessed by performing qRT-PCR using a SYBR Green PCR kit (TRAN, AQ602-24) on a CFX96 real-time PCR system (Bio-Rad, United States). All primer sequences were synthesized by Tsingke Biotechnology (Beijing, China). The primer sequences are shown in the **Supplemental material**.

### Statistical analysis

Statistical analyses were performed with Prism 8.0 software (GraphPad, La Jolla, CA, United States). All data were presented as the mean ± standard error of the mean (SEM).

Comparisons between two groups were analyzed by one-way ANOVA followed by the Tukey post-test. Statistical significance was accepted at *p <*0.05.

## Results

### GEO information

In the present study, four GEO datasets (GSE151839, GSE118553, GSE44000, and GSE122063) were loaded. The information from the four datasets is summarized in **Table 1**. For GSE151839 and GSE118553, we constructed a gene coexpression network based on WGCNA. GSE44000 and GSE122063 were applied to validate the differentially expressed genes (DEGs) analysis.

**Table 1:** Summary of those four GEO datasets involving OB and AD patients.

ID	GSE number	Platform	Samples	Source types	Disease
1	GSE151839	GPL570	10 patients and 10 controls	Subcutaneous adipose tissue	OB
2	GSE118553	GPL10558	52 patients and 27 controls	Cortex tissue	AD
3	GSE44000	GPL6480	7 patients and 7 controls	Subcutaneous adipose tissue	OB
4	GSE122063	GPL16699	12 patients and 11 controls	Cortex tissue	AD

### Identification of critical modules in OB and AD

**Figure 1:**
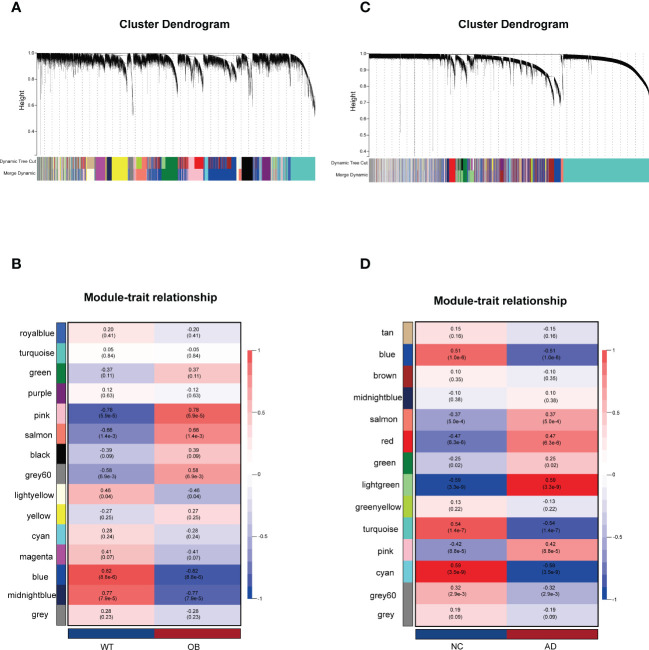
Weighted gene co-expression network analysis (WGCNA). **(A)** The Gene clustering tree (dendrogram) in OB. **(B)** Module–trait relationships in OB. Each cell contains the corresponding correlation and *p*-value. **(C)** The Gene clustering tree (dendrogram) in AD. **(D)** Module–trait relationships in AD. Each cell contains the corresponding correlation and *p*-value. OB, obesity; AD, Alzheimer’s disease.

A total of 15 modules were detected in GSE151839 according to the WGCNA and labeled with a unique color. To assess the correlation between modules and OB, a heat map was drawn about MEs and sample traits using the Pearson correlation coefficient (**Figures 1A, B**). The results showed that five modules were significantly associated with OB and were selected as OB-related modules (pink module: r = 0.78, *p* = 5.9e−5; salmon module: r = 0.66, *p* = 1.4e−3; gray60: r = 0.58, *p* = 6.9e−3; blue module: r = −0.82, *p* = 8.8e−6; midnightblue module: r = −0.77, *p* = 7.9e−5). Three modules (the pink, salmon, and gray60 modules) were positively related to OB, while the other two modules (the blue and midnightblue modules) were negatively related to OB. Likewise, a total of 14 modules were identified in GSE118553. Four modules were closely correlated with AD and were selected as AD-related modules (blue module: r = −0.51, *p* = 1.0e−6; turquoise module: r = −0.54, *p* = 1.4e−7; cyan: r = −0.59, *p* = 3.5e−9; lightgreen module: r = 0.59, *p* = 3.3e−9). Lightgreen module was the only one positively related to AD. Three modules (the blue, turquoise, and cyan modules) were negatively related to AD (**Figures 1C,D**). Then the above modules were remained for further analysis.

### Function annotation of the critical modules

**Figure 2:**
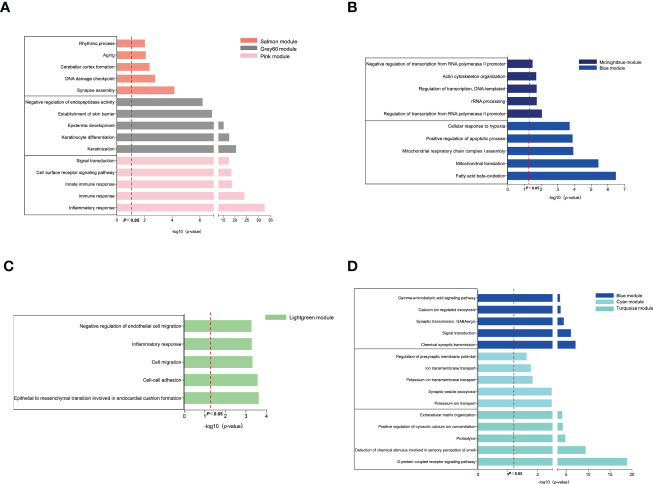
GO enrichment analysis of the modular genes. **(A)** The GO biological process analyses of three positive OB-related modules. **(B)** The GO biological process analyses of two negative OB-related modules. **(C)** The GO biological process analyses of one positive AD-related modules. **(D)** The GO biological process analyses of three negative AD-related modules. OB, obesity; AD, Alzheimer’s disease; GO, gene ontology.

Next, we investigated the biological function and pathways of genes from critical modules and their correlation with OB and AD, respectively by GO analysis. For the OB database, **Figure 2A** shows that genes from positive OB-related modules were enriched in inflammatory/immune response, keratinization, and synapse assembly, respectively. As shown in **Figure 2B**, genes from negative OB-related modules were related to fatty acid beta-oxidation, mitochondria, and RNA polymerase II, respectively. These results illustrated that OB was linked to an inflammatory/immune response and mitochondrial dysfunction.

Regarding the AD database, **Figure 2C** reveals that genes from positive AD-related modules were involved in the processes of the inflammatory response, including cell–cell adhesion and cell migration. **Figure 2D** indicates that genes from negative AD-related modules were enriched in the G-protein-coupled receptor signaling pathway, ion transport-related, and chemical synaptic transmission, respectively. However, mitochondria are fundamental to ion transport and synaptic transmission. Previous studies reported that mitochondrial dysfunction was believed to be an important contributor to the pathogenesis of AD (20, 21). These results demonstrated that AD was related to inflammation, ion transport dysfunction, and synaptic dysfunction.

### The common gene functional annotation in OB and AD

**Figure 3:**
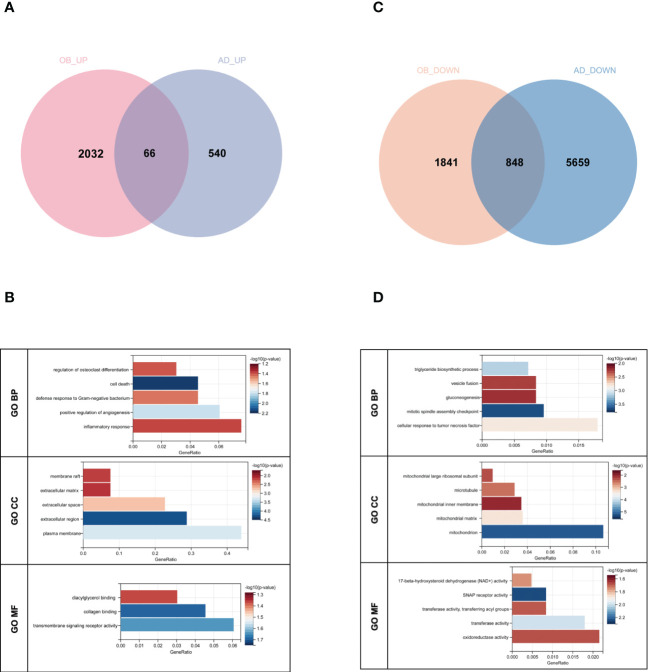
Venn diagram and GO enrichment analysis. **(A)** The shared genes between positive OB related and AD related modules. **(B)** GO analysis of shared genes between positive OB related and AD related modules. **(C)** The shared genes between negative OB related and AD related modules. **(D)** GO analysis of shared genes between negative OB related and AD related modules. OB, obesity; AD, Alzheimer’s disease.

There were 66 genes and 848 genes shared in positive and negative related modules of OB and AD, respectively, which were named gene sets 1 (GS1)-UP (**Figure 3A**) and GS1-DOWN (**Figure 3C**) and strongly linked to the pathogenesis of OB and AD. To understand the potential effects of GS1, we conducted GO analysis. As for GS1-UP, the results showed that the biological processes (BP) changed in the positive regulation of angiogenesis and inflammatory-related processes (inflammatory response, defense response to Gram-negative bacteria, cell death, and regulation of osteoclast differentiation). Cell component (CC) was mainly associated with the cell’s outer membrane (extracellular region, plasma membrane, extracellular space, membrane raft, and extracellular matrix). In terms of molecular functions (MF), only three pathways (transmembrane receptor activity, collagen protein binding, and diacylglycerol binding) were significantly enriched (*p*-value <0.05) (**Figure 3B**). Regarding GS1-DOWN, changes in BP are mainly associated with the mitotic spindle assembly checkpoint, the triglyceride biosynthetic process, the cellular response to tumor necrosis factor, vesicle fusion, and gluconeogenesis. These changes in CC were notably focused on enrichment of the mitochondrion (mitochondrial matrix, mitochondrial large ribosomal subunit, and mitochondrial inner membrane) and microtubule. In the MF section, GS1-DOWN was principally enriched in receptor activity (SNAP receptor activity) and enzymatic activity (NAD+ activity, transferase activity, and oxidoreductase activity) (**Figure 3D**).

Taken together, inflammation and mitochondrial dysfunction may be shared pathologies in obesity and AD patients.

#### PPI network establishment and analysis of hub genes

**Figure 4:**
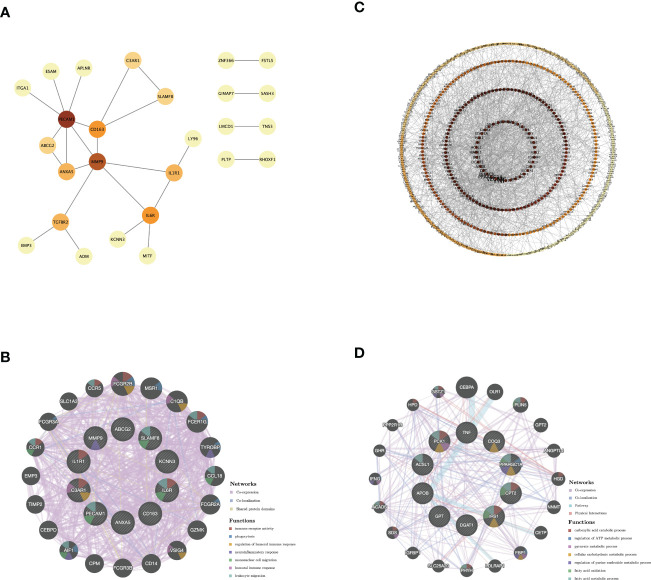
PPI network and co-expression network of hub genes. **(A)** PPI network diagram of GS1-UP. **(B)** GeneMANIA analysis of hub genes and their co-expression genes in GS1-UP. **(C)** PPI network diagram of GS1-DOWN. **(D)** GeneMANIA analysis of hub genes and their co-expression genes in GS1-DOWN. GS1, gene set 1.

The PPI network of genes from GS1-UP with a confidence score >0.4 was constructed to represent protein interactions through STRING and Cytoscape. There were 26 nodes and 26 edges (**Figure 4A**). Then hub genes were calculated *via* cytoHubba, including C3AR1, SLAMF8, ABCG2, ANXA5, CD163, IL1R1, MMP9, IL6R, PECAM1, and KCNN3. Next, the GeneMANIA database was used to analyze the co-expression network and related functions of these genes. These genes showed a network with a co-expression of 93.5% and a co-localization of 4.32%. The function of these genes is mainly involved in immunity and inflammation, particularly the neuroinflammatory response (**Figure 4B**). Similarly, the PPI network of genes in GS1-DOWN was established, which contained 639 nodes and 1,463 edges and selected hub genes, including PPARGC1α, DGAT1, CPT2, COQ3, TNF, PCK1, APOB, ACSL1, GPT, and IRS1 (**Figure 4C**). Then the GeneMANIA database showed that the network of hub genes had a co-expression of 74.52% and a co-localization of 17.1%. These hub genes were associated with metabolic and ATP processes (**Figure 4D**). These results emphasized the important role of inflammation and immune and metabolic pathways in these two diseases.

### Validation of genes in OB and AD

**Figure 5:**
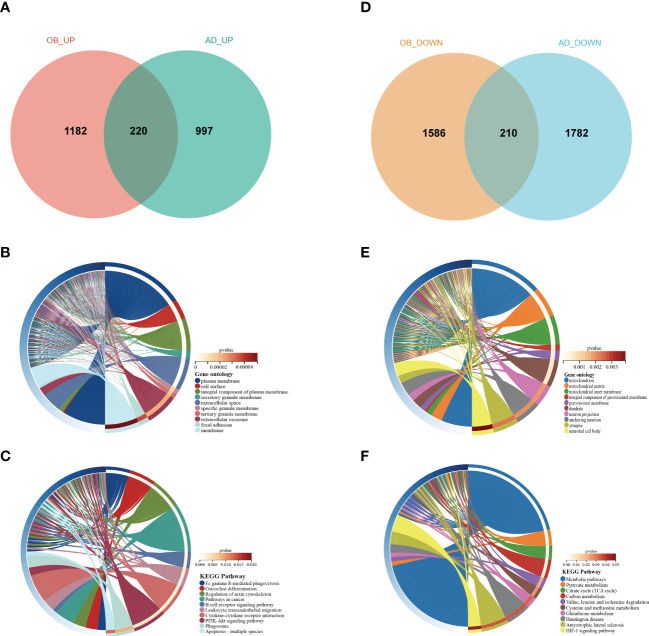
Venn diagram and enrichment analysis of the common DEGs. **(A)** The Venn diagram of the upregulated genes in GSE44000 and GSE122063. **(B)** The GO biological process analyses of common-upregulated genes. **(C)** The KEGG pathway of common-upregulated genes. **(D)** The Venn diagram of the downregulated genes in GSE44000 and GSE122063. **(E)** The GO biological process analyses of common-downregulated genes. **(F)** The KEGG pathway of common-downregulated genes. GO, gene ontology; DEGs, differentially expressed genes.

To validate our results, we selected two other datasets, GSE44000 and GSE122063, to analyze differential genes. For GSE44000, we obtained 3,178 DEGs, including 1,382 upregulated genes and 1,796 downregulated genes. In GSE122063, we obtained 3,209 DEGs, including 1,217 upregulated genes and 1,992 downregulated genes. There were 220 common upregulated genes and 210 common downregulated genes that overlapped, which were respectively defined as gene sets 2 (GS2)-UP and GS2-DOWN (**Figures 5A, D**). For GS2-UP, GO CC analysis was mainly enriched in plasma membrane, cell surface, and an integral component of the plasma membrane (**Figure 5B**). The major KEGG pathways were FCγR-mediated phagocytosis, osteoclast differentiation, and leukocyte transendothelial migration (**Figure 5C**). For GS2-DOWN, GO CC results showed these genes were chiefly involved in the mitochondrion, mitochondrial matrix, and mitochondrial inner membrane (**Figure 5E**). The KEGG analysis showed that these genes were primarily related to metabolic pathways, pyruvate metabolism, and the citrate cycle (**Figure 5F**). These results were highly consistent with the results in **Figure 3**.

Then, we found six overlapped genes in hub genes from GS1 and GS2, which could be divided into two categories: upregulated hub genes including MMP9 (Matrix Metallopeptidase 9), PECAM1 (platelet endothelial cell adhesion molecule-1), C3AR1 (Complement C3a Receptor 1), and IL1R1 (IL-1 receptor type 1), and downregulated hub genes PPARGC1α (PPARG Coactivator 1 Alpha) and COQ3 (Coenzyme Q3, Methyltransferase).

### Validation of candidate targets in animal models

**Figure 6:**
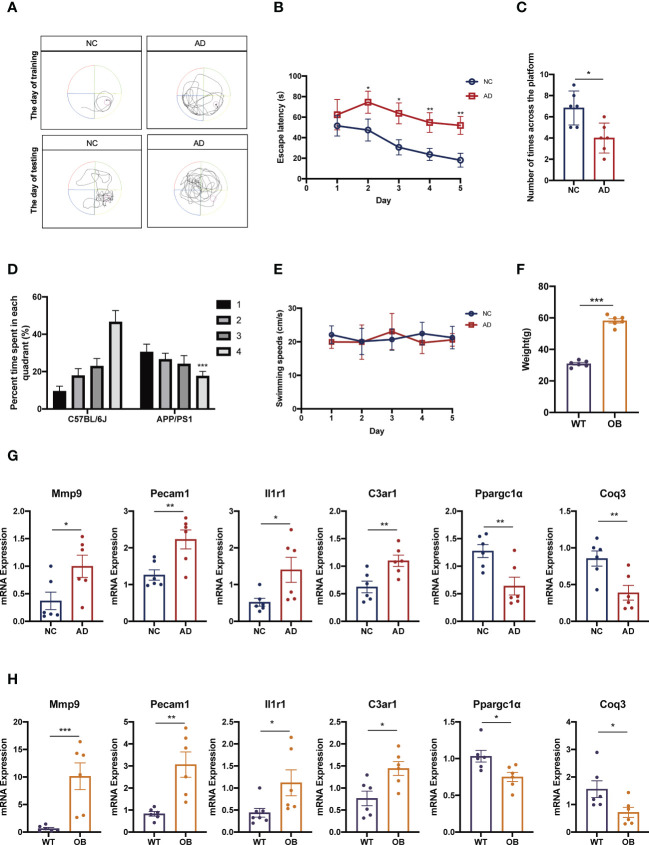
Confirmation of the different expression of candidate targets in animal models. **(A–E)** AD mice were subjected to hippocampal-dependent cognitive testing using the MWM. Data in **(A, B)** show the MWM training and data in **(C, D)** show the MWM testing. Data in **(E)** show mice swimming speeds. **(F)** Weight in in WT and OB mice. **(G)** The expressions of Mmp9, Pecam1, C3ar1, Il1r1, Ppargc1α, and Coq3 were analyzed by qPCR analysis in cortex tissues from AD and NC mice. **(H)** The expressions of Mmp9, Pecam1, C3ar1, Il1r1, Ppargc1α, and Coq3 were analyzed by qPCR analysis in subcutaneous adipose tissues from OB and WT mice. MWM, Morris water maze; WT, wild type; OB, obesity; NC, negative control; AD, Alzheimer’s disease. *p <0.05, **p <0.01, ***p <0.001.

We next verified the seven mentioned genes, respectively, in AD and OB animal models. Regarding the AD animal model, results from the MWM demonstrated that learning and memory ability were significantly decreased in the AD animal model as compared with that in the control mice on the training and testing days. The AD mice had a reduction period to stay in the quadrant where the platform was located and declined the number of times that mice crossed the platform (**Figures 6A–E**). OB mice developed significant obesity in comparison with wild-type (WT) mice (**Figure 6F**).

Finally, we performed quantitative RT-qPCR of these genes both in OB and AD mice. Our results showed that the expression levels of Mmp9, Pecam1, C3ar1, and Il1r1 in AD mice were significantly higher than those in control mice (**Figure 6G**). And the expressions of these genes in OB mice were also higher than those in WT mice (**Figure 6H**). About the expressions of Ppargc1α and Coq3, OB and AD mice showed significantly lower levels accompanied by WT and control mice (**Figures 6G, H**).

## Discussion

Various studies have suggested that mid-life obesity is a risk factor for later-life dementia, Parkinson’s disease, and Alzheimer’s disease (9, 22, 23). Particularly, obesity increases the risk of Alzheimer’s disease by 35% (24). Obesity and Alzheimer’s disease might have overlapping pathogenic pathways, particularly inflammation and mitochondrial dysfunction. However, it is still unclear how peripheral mitochondrial dysfunction and inflammation lead to brain mitochondrial dysfunction and neuroinflammation.

On the one hand, in obesity conditions, the hypertrophic adipose tissue secretes several pro-inflammatory adipokines, generating low-grade chronic inflammation (25). These processes affect not only adipose tissue but also put the brain in a low-grade chronic inflammatory state with activation of endothelial cells and glial cells (26, 27). In addition, recent evidence indicates that inflammation is recognized as a key component in Alzheimer’s disease pathogenesis (28). The pro-inflammatory cytokines can cross and disrupt the blood–brain barrier (BBB) (29–32). Central inflammation is exacerbated by a compromised BBB, contributing to disease progression in Alzheimer’s disease (33). In our study, we identified four hub genes (MMP9, PECAM1, C3AR1, and IL1R1) that were upregulated in all datasets. These genes are almost associated with inflammation and immune. Among these, MMP9 is involved in complications of obesity or metabolic syndrome through the breakdown of extracellular matrix (ECM) molecules (34). MMP9 may cause severe BBB disruption, resulting in enhanced inflammatory diseases of the central nervous system (35). Next, expression of Pecam1 was significantly upregulated and correlated strongly with body weight in diet-induced obese (DIO) mice (36). PECAM1 was also shown to be involved in the pathogenesis of Alzheimer’s disease *via* promoting neuroinflammation (37). Third, overexpressing C3aR could exacerbate obesity and other metabolic dysfunctions (38). The heightened C3a/C3aR signaling through endothelial cells promoted a series of inflammatory reactions, and BBB dysfunction contributes to overall neuroinflammation in aging and neurodegenerative disease (39). Fourth, IL-1R1 regulates the inflammatory response through agonistic and antagonistic modulation of cytokine activity. The IL-1 inflammatory cytokine is closely related to rheumatoid arthritis, type 2 diabetes mellitus, obesity, cancer, and neurodegenerative diseases (40). By further verification, we found that four hub genes were highly expressed both in the mice model of obesity and Alzheimer’s disease. Overall, inflammation may play the role of a bridge between obesity and Alzheimer’s disease, and the four hub genes above (MMP9, PECAM1, C3AR1, and IL1R1) are critical to this.

On the other hand, mitochondrial dysfunction has been observed in AD as well as obese individuals (41, 42). Mitochondrial morphological changes in presynaptic neurons impair synaptic homeostasis and may, therefore, lead to neurodegeneration (43). Obesity consistently results in mitochondrial dysfunction (44). Meanwhile, the mitochondrial impairment of obesity is able to stimulate the production of reactive ROS further to promote inflammation, thereby accelerating Alzheimer’s disease progression (45). We selected two hub genes (PPARGC1α and COQ3) that were downregulated in all datasets and were closely related to mitochondrial function. Downregulation of PPARGC1α impairs mitochondrial biogenesis and oxidative metabolism in obesity (46). PPARGC1α expression has been reported to be altered in neurodegenerative disorders, leading to mitochondrial defects and increased ROS levels; increasing its levels results in reductions in Alzheimer’s disease pathology and improvements in behavior (47). COQ3 is thus far unavailable in obesity and AD. COQ3 is a critical component of the electron transport pathways of both eukaryotes and prokaryotes, which are in the mitochondria’s inner membrane. Thus, obesity leads, at least in part, to the onset of AD by compromising mitochondrial function, while PPARGC1α and COQ3 play key roles in this process.

In conclusion, we illustrated the possible mechanism of AD secondary to OB *via* novel bioinformatic tools and approaches. Meanwhile, we revealed that the increased inflammation/immune response and mitochondrial dysfunction in OB might be an essential susceptible factor for AD and identified novel gene candidates (MMP9, PECAM1, C3AR1, IL1R1, PPARGC1α, and COQ3) who could be used as biomarkers or as potential therapeutic targets.

## Data availability statement

The datasets presented in this study can be found in online repositories. The names of the repository/repositories and accession number(s) can be found in the article/**Supplementary Material**.

## Ethics statement

The animal study was reviewed and approved by the Laboratory Animal Ethical Committee, Tianjin Medical University Chu Hsien-I Memorial Hospital.

## Author contributions

TL designed and conducted the whole research. TL and JQ applied for the GEO dataset analysis of AD. CX and TF applied for the GEO dataset analysis of OB. TL completed the data analysis and animal experiments,and drafted the manuscript. BS and LC revised and finalized the manuscript. All authors contributed to the article and approved the submitted version.
