# Supplementary material for: Exploring the common gene signatures and pathogeneses of obesity with Alzheimer’s disease via transcriptome data
Source: Front Endocrinol (Lausanne). 2022 Dec 9;13:1072955. doi: 10.3389/fendo.2022.1072955 (PMC9780446; doi:10.3389/fendo.2022.1072955)
Supplement: Supplementary file 1 [file Table_1.docx]

| **Gene name** | **Forward** | **Reverse** |
| --- | --- | --- |
| Mus β-actin | 5-AAGACCTCTATGCCAACACAG-3 | 5-GGAGGAGCAATGATCTTGATC-3 |
| Mus Mmp9 | 5-GCAGAGGCATACTTGTACCG-3 | 5-TGATGTTATGATGGTCCCACTTG-3 |
| Mus Il1r1 | 5-GTGCTACTGGGGCTCATTTGT-3 | 5-GGAGTAAGAGGACACTTGCGAAT-3 |
| Mus C3ar1 | 5-TCGATGCTGACACCAATTCAA-3 | 5-TCCCAATAGACAAGTGAGACCAA-3 |
| Mus Pecam1 | 5-CTGCCAGTCCGAAAATGGAAC-3 | 5-CTTCATCCACCGGGGCTATC-3 |
| Mus Ppargc1a | 5-TATGGAGTGACATAGAGTGTGCT-3 | 5-CCACTTCAATCCACCCAGAAAG-3 |
| Mus Coq3 | 5-ACACGGGACCATGTGCTTTAG-3 | 5-GCTGTCAACTGTTGTTTGTGAAG-3 |

Supplemental The sequences of primers for RT-qPCR analysis of the hub genes
